# Proximal and Distal Predictors of the Spider Monkey’s Stress Levels in Fragmented Landscapes

**DOI:** 10.1371/journal.pone.0149671

**Published:** 2016-02-22

**Authors:** José D. Ordóñez-Gómez, Jurgi Cristóbal-Azkarate, Víctor Arroyo-Rodríguez, Ana M. Santillán-Doherty, Ricardo A. Valdez, Marta C. Romano

**Affiliations:** 1 Posgrado en Ciencias Biológicas, Universidad Nacional Autónoma de México, Mexico City, Mexico; 2 Division of Biological Anthropology, University of Cambridge, Cambridge, United Kingdom; 3 Instituto de Investigaciones en Ecosistemas y Sustentabilidad, Universidad Nacional Autónoma de México, Morelia, Michoacán, Mexico; 4 Neurociencias, Instituto Nacional de Psiquiatría Ramón de la Fuente Muñiz, Mexico City, Mexico; 5 Departamento de Fisiología, Biofísica y Neurociencias, Centro de Investigación y Estudios Avanzados, Mexico City, Mexico; Università degli Studi di Napoli Federico II, ITALY

## Abstract

The rapid loss, fragmentation and degradation of tropical forests threaten the survival of many animal species. However, the way in which these phenomena affect animal health has been poorly explored, thus limiting the design of appropriate conservation strategies. To address this, here we identified using linear mixed models the effect of proximal (diet, activity pattern, hunting and logging) and distal (sum of the basal areas of fruiting-tree species [SBAFS], landscape forest cover and degree of forest fragmentation) variables over fecal glucocorticoid metabolite (fGCM) levels–hormones associated with animal health and fitness–of six groups of spider monkeys (*Ateles geoffroyi*) inhabiting six landscapes with different spatial structures in Mexico. Proximal variables showed a stronger predictive power over fGCMs than distal. In this sense, increases in travel time, the occurrence of hunting, and reductions in rest time and fruit consumption resulted in higher fGCM levels. Regarding distal variables, increases in SBAFS were negatively related to fGCM levels, thus suggesting that food scarcity increases stress hormone levels. Nevertheless, contrary to theoretical expectations, spider monkeys living in smaller tracts of forest spent less time travelling, but the same time feeding on fruit as those in more forested areas. The lower net energy return associated with this combination of factors would explain why, contrary to theoretical expectations, increased forest cover was associated with increased levels of fGCMs in these groups. Our study shows that, at least in the short term, spider monkeys in fragmented landscapes do not always present higher levels of stress hormones compared to those inhabiting continuous forest, and the importance of preserving fruit sources and controlling hunting for reducing the levels of stress hormones in free ranging spider monkeys.

## Introduction

Land-use change has converted more than three quarters of the terrestrial biosphere into human-modified landscapes [1,2]. Because biodiversity is often threatened in these emerging landscapes, an increasing number of studies have been directed to better understand the response of species to such landscape changes and inform conservation plans (reviewed in [3–5]). The majority of these efforts have been focused on assessing the impact of forest patch and landscape spatial attributes on animal behavior [6,7], and on other attributes of animal and plant populations [8,9] and assemblages [10–12]. However, the complexity of the effects set off by habitat loss and fragmentation over the living conditions of animals hampers our ability to pinpoint which are the proximal factors (e.g., daily activity patterns, diet, intra and intergroup conflicts) that lead to the negative effects of these broad phenomena over species’ demography, and how these are in turn associated with distal effects, such as landscape metrics. This is further complicated by the slow life histories of many animal taxa such as primates, and by the fact that the response of species to changes in habitat spatial metrics (e.g., forest loss and fragmentation) depends on the spatial scale (i.e., landscape size) at which these metrics are measured (i.e., the so-called “scale of effect”; sensu Jackson and Fahrig [13]).

The study of glucocorticoids (e.g., cortisol, corticosterone) can help us to assess the effects of habitat perturbation on wildlife’s energetic physiology and fitness. These hormones are secreted by the adrenal glands in response to stressful challenges to the organism’s homeostasis, their principal effect being the mobilization of energy reserves to overcome the stressor and the inhibition of non-vital functions [14–16]. This response is therefore adaptive, but higher levels of glucocorticoids (GC) are nonetheless indicators that organisms have to cope with a certain challenge using resources that could be allocated for maintenance and reproduction [17]. Accordingly, chronically elevated levels of GCs have been associated with pregnancy loss, lower birth weight and immunosuppression [18,19], and therefore, these hormones have been proposed as biomarkers of population health [20].

Due to their large size, diurnal habits and adaptability to human presence, it is relatively easy to follow arboreal primates and register their diet and behavior. This makes them a good model for identifying the proximal causes of stress in wildlife, and determining how these are in turn associated with broader phenomena, such as reduced resource availability and changes in landscape structure (e.g., forest cover and degree of forest fragmentation). Overall, studies show that primates living in smaller forest fragments have higher levels of GCs than those living in larger tracts of forest (e.g., *Piliocolobus tephrosceles*: [21]; *Alouatta pigra*: [22]; *Eulemur collaris*: [23]). Dunn et al. [24] related the higher levels of GCs in feces of a group of howler monkeys (*Alouatta palliata*) living in a small forest fragment to a lower availability of fruit, which in turn forced them to travel more and consume larger quantities of energy-poor leaves. Studies of *Pan troglodytes schweinfurthii* [25] and *Cercopithecus mitis* [26,27] have also related higher GC levels with food shortage and higher metabolic demands. In addition to this, other proximal factors such as human presence [28,29] and direct anthropogenic disturbances (e.g., logging, hunting) [30] may also impact GC levels in primates. Nevertheless, to date, no environmental physiology study has yet assessed the effect of landscape spatial metrics on stress levels of primates, despite evidence showing that landscape attributes like forest cover and forest fragmentation are important predictors of their occurrence [11], behavior [7] and population characteristics [31] in fragmented tropical landscapes.

Here, we used for the first time a patch-landscape approach (sensu Arroyo-Rodríguez & Fahrig [32]) to identify the main predictors of stress levels in spider monkeys (*Ateles geoffroyi*) living in human-modified landscapes. In particular, we analyzed what set of proximal (activity pattern, diet and direct anthropogenic disturbances) and distal (sum of the basal areas of fruiting-tree species [SBAFS] and landscape structure) variables better predicted the levels of fecal glucocorticoid metabolites (fGCMs) in six groups of spider monkeys inhabiting landscapes with different degree of human perturbation in the Lacandona rainforest, Mexico. Given the highly energetic demands of spider monkeys [33,34] and the patchy distribution of fruit in space, we predicted that an increase in fruit consumption (i.e., proximal predictor), forest cover and density of fruit sources (i.e., distal predictors) would result in lower levels of fGCM. Also, because logging and hunting (i.e., direct anthropogenic disturbances) have a direct effect on the survival of arboreal animals, we predicted that the occurrence of these activities would increase fGCM levels. This is a timely study for the conservation of *A*. *geoffroyi*, given that this large-bodied and highly frugivorous Neotropical primate [35] is considered one of the most sensitive species to habitat transformation [36–38]. In fact, *A*. *geoffroyi* is classified as “Endangered” in the IUCN red list, and it is estimated that the populations have declined by as much as 50% over the course of the past 45 years [39].

## Materials and Methods

### Ethics Statements

This research was undertaken in accordance with the ethical and legal requirements of the Secretaría de Medio Ambiente y Recursos Naturales (SEMARNAT) of Mexico, and was authorized by permit number SGPA/DGVS/04045/13. It also complied with the protocols approved by the Ethical Committee of the Instituto Nacional de Psiquiatría Ramón de la Fuente Muñiz (approval no. 3330B) of Mexico, and adhered to the American Society of Primatologists Principles for the Ethical Treatment of Non-Human Primates. The owners of the forest fragments granted us the permission to conduct this study on their land.

### Study area

We conducted the fieldwork for this study in the Lacandona rainforest, in southern Chiapas, Mexico. This region presents two well-defined seasons: a dry season from January to May, and a rainy season from June to December. The average annual rainfall is 2881 mm, with the highest concentration of rainfall occurring between June and September (range: 423–511 mm/mo) and the lowest between February and April (range: 46–61 mm/mo). During the dry season the average monthly temperature is 26.3°C (range: 22–28°C) while during the rainy season it is 23.5°C (range: 20–25°C) (Comisión Federal de Electricidad, Mexico). Although there are no long-term published records on changes in fruit availability for our study region, a recent study of the seed rain produced by spider monkeys in latrines located beneath 60 sleeping sites in the region [40,41] suggests that in the Lacandona region the production of fleshy fruit (main food item for *A*. *geoffroyi* [33,34]) is higher during the rainy season than during the dry season, which has been associated with the fact that spider monkeys tend to consume smaller quantities of fruit during this period [42].

In terms of habitat configuration, our study region presents two well-distinguished areas: (i) the Montes Azules Biosphere Reserve (MA), which encompasses 331,200 ha of continuous forest; and (ii) the Marqués de Comillas Region (MCR), which is comprised of 203,999 ha of fragmented forest embedded in a matrix dominated by agricultural lands, cattle pasture, and human settlements. Deforestation in MCR started in the 1970s, but it was particularly accelerated and extensive between 1984 and 1996 [43], and as a result the MCR has lost approximately 60% of its original forest cover.

### Studied spider monkey groups

We collected fecal samples and analyzed the behavior of spider monkeys (*Ateles geoffroyi*) belonging to six different groups living in separate sites (F1, F2, F3, F4, F5 and MA) characterized by landscapes of different structure. Group MA lived in the Montes Azules Biosphere Reserve (16°07’N, 90°56’W), while the other five groups inhabited different forest fragments within the Marqués de Comillas region (F1: 16°15’N, 90°50’W; F2: 16°18’N, 90°40’W; F3: 16°17’N, 90°50’W; F4: 16°20’N, 90°51’W; F5: 16°20’N, 90°48’W). Group MA was composed of 26 individuals (11 females, 4 males, 6 juveniles and 5 infants), Group F1 of 30 individuals (13 females, 5 males, 4 juveniles and 8 infants), Group F2 of 30 individuals (13 females, 4 males, 6 juveniles and 7 infants), Group F3 of 27 individuals (12 females, 6 males, 3 juveniles and 6 infants), Group F4 of 22 individuals (8 females, 5 males, 3 juveniles and 6 infants) and finally Group F5 of 23 individuals (6 females, 9 males, 5 juveniles and 3 infants).

### Collection of proximal variables

Before the start of the data collection, and with the aim of habituating the study groups, identifying the group members and locating their most frequent travel routes, we followed each of the six study groups for *ca*. four hours every two weeks during two different periods (February-August 2012 and January-February 2013), for a total of 421 h (range: 65–72 h per group). We determined that the study groups were habituated to our presence when they stopped threatening (e.g., shaking branches and vocalizing against us) and/or paying attention to us (e.g., gazing). We also identified as a sign of habituation the fact that females would sometimes allow their infants to get very close to us (*ca*. 3–6 m).

We conducted our study during the dry season of 2013 (March to May), because studies with spider and howler monkeys suggest that the effects of forest loss and fragmentation on the behavior [24,42] and stress levels [24] of primates are more marked during periods of seasonal reductions in the availability of fleshy fruit. We studied the daily activity pattern and diet, and collected fecal samples from each study group once every three weeks (i.e., sampling round) for three consecutive days from 0700 to 1530 h. In total we conducted three sampling rounds per group. To avoid the potential effect of age on monkey’s behavior and hormone levels we sampled only adult individuals. Observations were carried out by two people (JDOG and a local field assistant), following a focal animal sampling method [44]. We switched focal animals randomly at 3-min intervals or whenever animals moved out of sight. Spider monkeys have high fission-fusion dynamics, therefore, to avoid biases due to sampling subgroups with adults of only one sex, during each fission event we selected a subgroup composed of adults of both sexes. The average number (±SD) of fission and fusion events per day was 1.278 (±0.712) and 0.759 (±0.671), respectively. We did not observe differences in the number of fission and fusion events per site (one-way ANOVA: F_5,48_ = 1.546, *P* = 0.193 for fission events; F_5,48_ = 0.712, *P* = 0.617 for fusion events). In total, we recorded 407 hours of focal observations (range per group = 66–69 h) (see further details in [7]).

We recorded the diet and activity pattern considering six mutually exclusive behavioral categories: (i) time resting (period of inactivity); (ii) time traveling (movement between tree crowns or within the crown of a tree that was not directly food related); (iii) time spent feeding on fruit; (iv) time spent feeding on leaves; (v) time spent feeding on wood; and (vi) other activities (e.g., intra and inter group aggression, grooming, aggression towards the observer). We did not consider the effect of ‘other activities’ for analysis because their occurrence can potentially be associated to social dynamics within the groups such as reproductive competition or conflict managements during fusion events, rather than to habitat effects. During each observation day we also recorded the occurrence of direct anthropogenic disturbances, such as logging and/or hunting in the vicinity of the study groups. We classified as: (i) ‘logging days’, days in which we heard tree saws or tree axes; (ii) ‘hunting days’, days in which we detected gun shots accompanied with barks of hunting dogs; and (iii) ‘no-disturbance days’, days in which we did not detect any signs of logging or hunting activities.

### Distal factors: landscape structure and food availability

The method that we followed to assess landscape spatial metrics has been described elsewhere [7]. Briefly, we used the method of minimum convex polygon to estimate the centroid of the activity area of each group based on the locations in which spider monkeys spent ≥30 min. Then, from this point we calculated the percentage of forest cover and the number of forest fragments within ten different-sized buffers (i.e., landscapes) to test the relative impact of forest loss and fragmentation on spider monkeys’ stress levels [32,45]. The scale of effect is related to spatial habitat use [13]. Accordingly, for the smallest buffer, we chose 50 ha as this is just below the 56 ha of the average spider monkey home range size reported for MA [42]. For the largest buffer, we chose 665 ha, which was the largest buffer that we could project without our six different landscapes started overlapping in space, loosing independence among samples. Using these two extreme values as reference, we then established eight additional buffers of incremental area (Table 1). After this, we calculated the Pearson correlation coefficient between each metric and the mean fGCM levels of each site, in order to identify the scale at which the strongest associations were presented (i.e., “scale of effect” [46]) (Table 1). We conducted all these analyses using a recent (April and May 2012) and high-resolution (2.5 × 2.5 m) SPOT satellite image and Quantum GIS 2.0.1 software.

**Table 1:** Pearson correlation coefficients and significant values (in parentheses) of the associations between landscape spatial metrics and the mean of log-transformed fecal glucocorticoid metabolite (fGCM) values of six groups of spider monkeys (*Ateles geoffroyi*) living in the Lacandona rainforest, Mexico.

Landscape metric/response variable	Size (ha) of local landscape*									
	50	84	126	177	237	305	382	468	562	665
Forest cover vs log (fGCM levels)	**0.592**	0.527	0.510	0.518	0.529	0.540	0.547	0.547	0.551	0.549
	(0.108)	(0.141)	(0.151)	(0.146)	(0.140)	(0.135)	(0.131)	(0.131)	(0.128)	(0.130)
Number of fragments vs log (fGCM levels)	**-0.558**	-0.247	-0.240	0.096	-0.037	-0.005	-0.176	-0.304	-0.050	-0.095
	(0.125)	(0.319)	(0.324)	(0.428)	(0.473)	(0.497)	(0.369)	(0.279)	(0.462)	(0.429)

*We indicate the highest coefficient values for each predictor in boldface.

To assess food availability within each site, we used the sum of the basal area of fruiting-tree species of which fruit is consumed by spider monkeys (SBAFS), as the basal area of a tree is a good proxy of the amount of fruit it can produce [47,48], and several studies report significant associations between this vegetation attribute and primate presence in forest fragments (e.g., *Cercocebus galeritus*: [49]; *A*. *palliata*: [50]). To calculate this, we randomly located 20 transects of 50 × 2 m within the activity area of each group, and we measured the diameter at breast height (dbh) of tree species with a dbh ≥ 10 cm. We then identified the tree species used by spider monkeys within these plots to calculate the SBAFS for each study group (S1 Table). The species used by spider monkeys for fruit consumption were identified combining the data from: (i) the present study, (ii) a year-round study conducted in the study region [34], and (iii) a meta-analysis study of the food species reported for *A*. *geoffroyi* in Mesoamerica [33].

### Fecal sample collection and fecal GC assay

While in the field, we opportunistically collected fecal samples throughout the day and immediately upon defecation (< 10 min), and we stored them in a cooler with frozen gel packs. We only collected samples if free of urine and other impurities, and from adult individuals. For each sample we registered location, date and time of collection, the sex of the individual the sample belonged to, and when female, whether she was lactating or non-lactating (pregnant and cycling females). Due to the large group sizes and fission-fusion dynamics of spider monkeys [51], we could not assign the identity of the individual who defecated to all samples. However, to reduce problems of data independence, during each observation day we collected only one sample per individual of the sampling subgroup. Back at the field station, every afternoon, we stored the samples at –20°C until their extraction (≤ 6 mo) in the Physiology Department of Centro de Investigación y de Estudios Avanzados, in Mexico City.

The protocols that we used to extract [52,53] and quantify [54] GC metabolites in feces have been previously used and validated to detect the activation of the HPA axis in response to stressful stimuli in spider monkeys (*Ateles geoffroyi*). Following the method described by Brown et al. [55] and modified by Brousset et al. [56], we dried out the samples at 65°C in a scientific oven (Precision Scientific 25EM) and pulverized and passed them through a sieve. Following this, we vortexed 0.40±0.01 g of each dried sample for 1 min in 5 mL of 80% ethanol and then placed them in a water bath at 80°C for 20 min. After the 20 min incubation period, we centrifuged the samples for 20 min at 460 × g and decanted the supernatant into a second tube. We then dried out the supernatants in a water bath at 36°C, added 0.3 mL ethanol and incubated the samples for 30 min at room temperature. Finally we centrifuged the samples for 20 min at 460 × g, and decanted the supernatants which were kept at –24°C until radioimmunoassay (RIA). Fecal extraction efficiency was (mean±SE) 66.4±1.1 CV% (*N* = 6), as measured by the recovery of ^125^I-cortisol.

We quantified GC concentrations in the samples with a solid phase ^125^I RIA method using cortisol CORT-CT2 CIS kits (Bio Internacional® B.P. 32-F91192 GIF-SUR-YVETTE CEDEX/France). The calibration range for assay was 0–2000 nmol l^-1^. We incubated the samples for 2 hr at 37°C and measured radioactivity using a Packard Cobra II® (Packard Cobra II, A Canberra Co. Meriden, CT) scintillation counter for gamma radiation. The kit presents a low cross- reactivity with corticosterone (2.5%) and cortisone (2.2%). We assessed all the extracts in duplicate in a total of seven assays. Intra- and inter-assay coefficients of variation were 7.8% and 8.3%, respectively. We performed parallelism by comparing the slope of a serial dilution curve of pooled spider monkey fecal extracts to the slope of the standard curve, difference not being significant (*t* = 0.356, *P* = 0.726, *N* = 10). The slope of standards spiked with diluted fecal extract exhibited high accuracy (*B* = 0.969, *R*^*2*^ **=** 0.992 *N* = 10, *P* < 0.001), indicating that the assay reliably measures fGCMs across its range of concentration.

### Statistical analyses

Spider monkeys present a very short food passage time (mean = 4.4 h, range: 2.75–7.75 h [57]), which results in that cortisol levels have been observed to peak in feces as little as 7–8 h after a stressful stimulus [52]. Accordingly, here we assessed the effect of proximal predictors on fGCM levels, by matching each individual’s fecal sample with: (i) the percentage of time the group to which that spider monkey belonged spent travelling, resting and consuming food items (fruit, leaves and wood); and (ii) the occurrence of logging or hunting in the vicinity the day in which the sample was collected. To assess the effect of distal predictors on fGCM levels, we matched the fGCM value of the fecal samples with the landscape spatial metrics (i.e., forest cover and number of forest fragments) and the SBAFS corresponding to the sites in which we collected the samples. Finally, we log transformed our response variable (fGCM levels) to achieve normal distribution.

We used the lmer function of the lme4 package [58] for R 3.2.2 to run linear mixed models (LMMs) to assess the effect of proximal and distal predictors on fGCM levels. We used the r.squaredGLMM function of the MuMln package [59] to calculate the coefficient of determination (*R*^2^) for each model. To reduce correlation and collinearity among predictors, we discarded variables that presented a Pearson correlation index > 0.7 and a variance inflation factor (VIF) > 4 (which indicate multicollinearity [60,61]). For calculating VIF, we used the function vif of the car package [62]. Because landscape spatial metrics and food availability (i.e., distal predictors) have been shown to be collinear with the daily activity pattern and diet (i.e., proximal predictors) of spider monkeys in our study region [34,42], we separately analyzed the impact of proximal and distal predictors on fGCMs. Given that we only collected one sample per individual per sampling day of the study subgroup, we controlled for pseudoreplication effects by specifying observation day (nested within sampling rounds) and samples (nested within sites) as random factors. To reduce the variability of fGCM levels due to factors not related to our independent variables, we used time of sample collection (AM or PM), sex, and lactating or non-lactating as control variables in all the models. We conducted multiple comparisons among the direct anthropogenic disturbances with the function ghlt of the package multcomp [63] for R 3.2.2 with *P* values adjusted using the Tukey method.

To select the most parsimonious models that best predicted the effect of predictor variables on fGCM levels, we used the Akaike’s information criterion (AIC). We ranked the models from the best (with lowest AIC) to the worst (with highest AIC), and considered the set of models with a difference in AIC (ΔAIC) < 2 from the top model as having equivalently strong empirical support and similar plausibility [61]. In order to check the assumptions of homogeneous and normally distributed residuals, we visually inspected Q-Q plots of residuals plotted against fitted values of each model.

Finally, we used the lmer function [58] to run a set of LMMs to analyze the effects of site on daily variation of activity pattern and diet of spider monkeys. We transformed percentages of the daily time spent in each time budget component (e.g., time travelling, time feeding on fruit) to proportions, and then, proportion data were arcsine-square-root transformed to meet normality assumptions. In each model we categorized site as a fixed factor, and sampling round as a random factor to account for the non-independence of repeated measures, and we applied a Bonferroni correction for multiple comparisons. We conducted *post hoc* pairwise comparisons between the study sites with the function lsmeans of the package lsmeans [64] with *P* values adjusted using the Tukey method. We carried out all analyses in R 3.2.2 [65].

## Results

We recorded the occurrence of hunting in site F2 and logging in site F3. Both types of disturbances were recorded every day during the second and the third sampling rounds (mean number of gun shots heard per day: 3.17, range: 2–5; mean duration of the logging activities per day: 1.86 h, range: 1.22–3.05 h) (Table 2). Forest cover was the landscape spatial metric that showed the strongest correlations with fGCM levels. The correlations of this metric were positive in all landscape sizes, being the strongest correlation in the 50-ha landscape size (*r* = 0.59). In the following landscape sizes forest cover showed lower correlations with gradual changes (range of *r*: 0.51–0.55). Opposite to forest cover, number of fragments showed negative correlations with fGCM levels (Table 2). This metric also showed its strongest correlation in the 50-ha landscape size (*r* = -0.56), and in the following landscape sizes the correlation values showed a strong decrease (Table 1). It is important to mention that although sites F4 and F5 showed the lowest values of forest cover, these sites presented high SBAFS (Table 2 and S1 Table).

**Table 2:** Variables used in linear mixed models to assess the effect of proximal and distal predictors of fecal glucocorticoid levels on six groups of spider monkeys (*Ateles geoffroyi*) living in the Montes Azules Biosphere Reserve (MA) and five sites (F1-F5) in the Lacandona rainforest, Mexico.

	MA	F1	F2	F3	F4	F5
***Characteristics of the study sites***						
**Distal predictors**						
Forest cover (%) in a 50-ha local landscape	100	98.2	62.0	51.5	34.1	21.9
Number of fragments in a 50-ha local landscape	0	1	1	2	1	4
SBAFS (m^2^) in 0.2 ha of sampling area per site^a^	7.6	7.1	4.2	4.7	5.9	6.3
**Number of spider monkey groups**^b^	> 5	3	2	2	1	1
**Site size (ha)**	330,000	1,125	460	141	67	28
***Characteristics of the study groups***						
**Proximal predictors (*N* = 54, 9 samples per group)**						
Mean percentage of time traveling (±SD)	25.4 (±5.5)	20.0 (±3.1)	19.8 (±4.6)	19.5 (±4.1)	14.6 (±3.4)	12.9 (±3.9)
Mean percentage of time resting (±SD)	39.9 (±6.4)	44.6 (±12.1)	50.2 (±9.4)	51.4 (±7.6)	54.7 (±4.7)	47.9 (±9.9)
Mean percentage of time feeding on fruit (±SD)	12.7 (±6.6)	24.2 (±15.3)	12.1 (±4.8)	11.8 (±3.9)	14.1 (±8.5)	17.9 (±14.9)
Mean percentage of time feeding on leaves (±SD)	2.7 (±1.6)	3.6 (±2.5)	3.4 (±4.2)	6.6 (±7.1)	10.5 (±4.7)	6.2 (±5.3)
Mean percentage of time feeding on wood (±SD)	12.7 (±6.8)	1.4 (±2.8)	1.0 (±1.5)	0.5 (±0.8)	0.32 (±0.65)	0.7 (±1.2)
Presence of direct anthropogenic disturbances^c^	No-disturbance	No-disturbance	Hunting	Logging	No-disturbance	No-disturbance
***Characteristics of the response variable* (*N* = 252)**						
**Mean (±SD) values of fGCM levels (nggn**^**-1**^**)**	255 ± 40	216 ± 19	339 ± 43	294 ± 46	134 ± 18	113 ± 12
**Feces collected**^d^ **(N)**	44	45	35	44	44	40

^a^Sum of basal areas of fruiting-tree species used by spider monkeys for fruit consumption.

^b^Number of groups living in the studied fragments and in MA (Montes Azules Biosphere Reserve).

^c^Distribution of collected samples across direct anthropogenic disturbances: No-disturbance = 188; Logging = 33; Hunting = 31.

^d^Distribution of collected samples across sexual status categories and collection time categories: Sexual status: Males = 82; Lactating female = 117; Non-lactating female = 53; Collection time: AM = 144; PM = 108.

**Fig 1:**
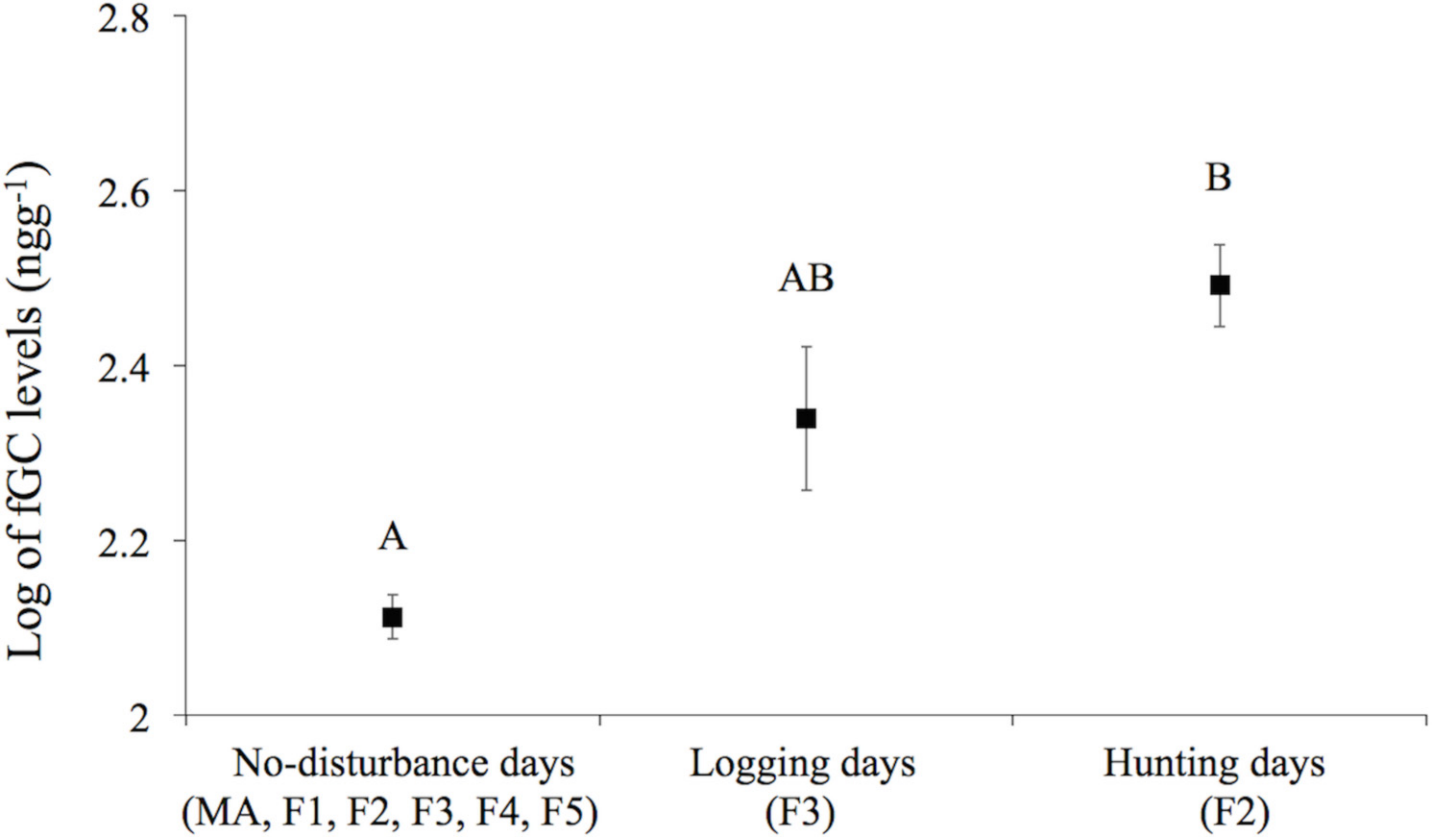
Mean (±SE) log fecal glucocorticoid metabolite (fGCM) levels of direct anthropogenic disturbances presented at six groups (MA, F1, F2, F3, F4, F5) of spider monkeys (*Ateles geoffroyi*) living in the fragmented Lacandona rainforest, Mexico. Letters indicate significant differences (*P* < 0.05) among direct anthropogenic disturbances.

The best-supported models that assessed the effects of proximal predictors on fGCM levels included: time traveling, time resting, direct anthropogenic disturbances, time feeding on fruit and time feeding on wood (S2 Table). Increases in travel time resulted in significantly higher levels of fGCM levels while the effect of rest time was the opposite (Table 3). The occurrence of hunting was associated with significantly higher fGCM levels than no-disturbance (Table 3 and Fig 1). Fruit consumption tended to decrease fGCM levels, although this effect did not reach significance (*P* = 0.07). Regarding distal predictors, the best-supported model included forest cover and SBAFS (S2 Table), but the variance explained by such fixed factors was notably lower than that explained by proximal predictors (Table 3). Increased forest cover was associated with significantly increased levels of fGCMs, while higher SBAFSs resulted in lower levels of stress hormones (Table 3). In all models we did not find significant differences among sexual status categories (Table 3).

**Table 3:** Results of the best-supported linear mixed models examining the effect of proximal and distal predictors on log-transformed fecal glucocorticoid levels of six groups of spider monkeys (*Ateles geoffroyi*) in the Lacandona rainforest, Mexico.

Predictor variables / Models	Estimate	SE/d.f. *	*t*/χ^2^**	*P* ***	VIF	AIC	M *R*^2^	C *R*^2^
***Proximal predictors***								
**TT+TR+TFF+TFW+DAD**						139.82	0.321	0.404
Intercept	2.779	0.317	8.773	**0.000**				
Time travelling	0.020	0.006	3.633	**0.001**	1.704			
Time resting	-0.013	0.004	-3.023	**0.004**	1.855			
Time feeing on fruit	-0.007	0.004	-1.842	0.070	1.785			
Time feeding on wood	-0.009	0.005	-1.771	0.081	1.323			
Direct anthropogenic disturbances		2	9.508	**0.001**				
Sexual status		2	0.268	0.875				
Collection time		1	1.759	0.185				
***Distal predictors***								
**Forest Cover+SBAFS**						165.7	0.113	0.381
Intercept	2.338	0.170	13.208	**0.000**				
Forest cover	0.005	0.001	3.648	**0.001**	1.384			
SBAFS	-0.072	0.032	-2.267	**0.028**	1.384			
Collection time		1	1.742	0.187				
Sexual status		2	0.143	0.931				

In all models, samples (nested within groups) and observation days (nested within sampling rounds) were specified as random factors. The variance inflation factor (VIF) is indicated for continuous variables. Marginal *R*^2^ (M *R*^2^) represents the variance explained by fixed factors, and conditional *R*^2^ (C *R*^2^) represents the variance explained by both fixed and random factors. TT = Time traveling, TR = Time resting, TFF = Time feeding on fruit, TFW = Time feeding on wood, DAD = Direct anthropogenic disturbances SBAFS = Sum of basal areas of fruiting-tree species used by spider monkeys for fruit consumption. Variables that significantly affected fGCM levels in boldface.

*For continuous variables we reported the standard error, and for categorical variables the degrees of freedom (d.f.).

**For continuous variables we reported the *t* value, and for categorical variables the χ^2^ value.

***For continuous variables we reported the *P* of the *t* value, and for categorical variables the *P* of the χ^2^ value.

**Fig 2:**
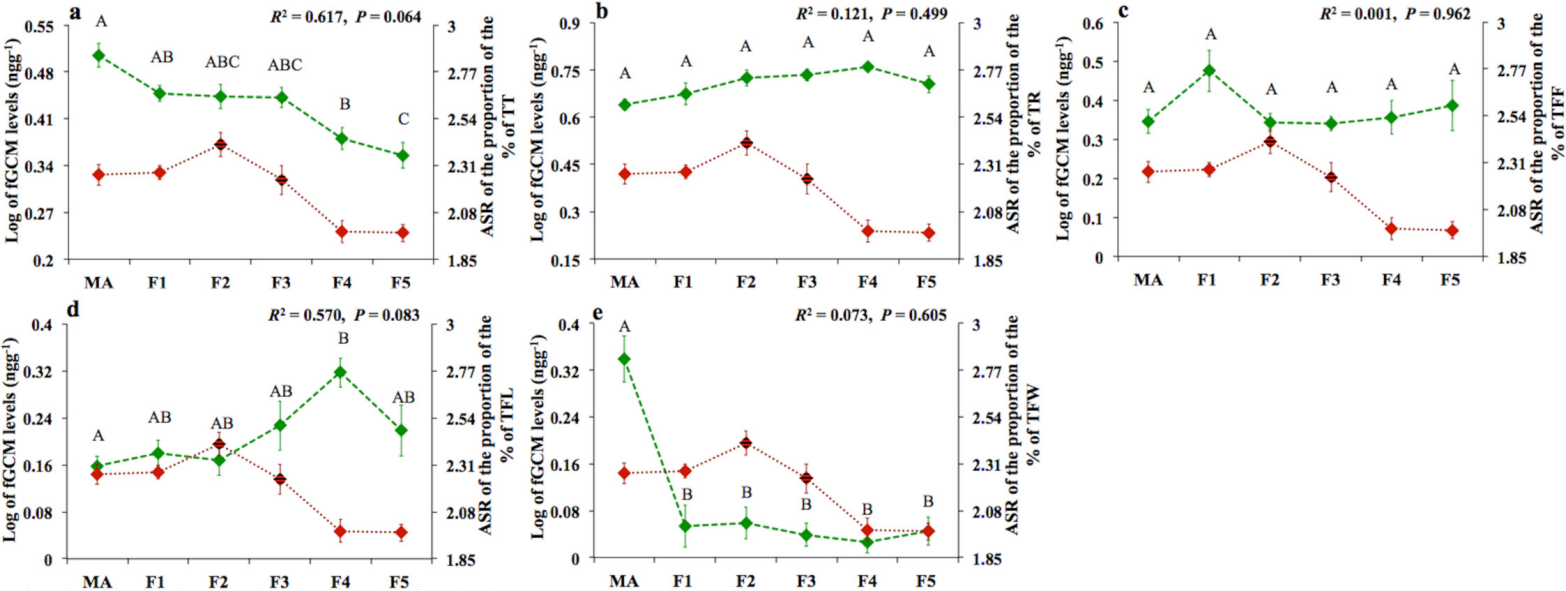
Relationships between means (±SE) of log-transformed fecal glucocorticoid metabolite (fGCM) levels (red) and means (±SE) of the arcsine-square-root (ASR) transformed of the proportion of the percentage of the daily time budgets (green) of six groups of spider monkeys (*Ateles geoffroyi*) living in the fragmented Lacandona rainforest, Mexico. TT = time traveling; TR = time resting; TFF = time feeding on fruit; TFL = time feeding on leaves; TFW = time feeding on wood. Letters indicate significant differences (*P* < 0.05) of daily time budgets among sites, and black lines in squares indicate the occurrence of direct anthropogenic disturbances within the sites.

We found significant effects of site in three of the five activity budgets we studied (Table 4). Spider monkeys spent more time traveling in MA than in F4 and F5, and in F1 than in F5 (Fig 2A). In the case of wood consumption, spider monkeys spent more time feeding on wood in MA than in all other sites (Fig 2E), and in the case of leave consumption spider monkeys spent more time feeding on leaves in F4 than in MA (Fig 2D). For time resting and feeding on fruit we found no significant effects (Table 4).

**Table 4:** Results of linear mixed models examining the differences of the daily activity pattern and diet among six groups of spider monkeys (*Ateles geoffroyi*) that inhabited different sites located in the Lacandona rainforest, Mexico.

Behavior	Site		
	χ^2^	d.f.	*P*
Time traveling	36.946	5	0.000*
Time resting	6.239	5	0.284
Time feeding on fruit	3.291	5	0.654
Time feeding on leaves	20.444	5	0.001*
Time feeding on wood	69.583	5	0.000*

*P < 0.01 i.e., significant value obtained after applying the Bonferroni correction for multiple tests.

## Discussion

Our study shows that fGCM levels of spider monkeys are affected by both proximal and distal variables. Consistent with our predictions, our results suggest that fruit consumption and a lower time spent on energetically costly activities (less traveling and more resting) result in lower levels of fGCMs (for examples with similar results see [24,26,66]), and that direct anthropogenic disturbances activate the stress response in spider monkeys [30]. Regarding distal predictors, we verified that fGCM levels are negatively related to SBAFS, thus suggesting that food scarcity within the sites increases stress levels [28,66,67]. However, contrary to our prediction, our results show a positive relationship between forest cover and fGCM levels, suggesting that spider monkeys inhabiting landscapes with low forest cover do not necessarily always experience higher levels of stress. Below, we elaborate on the potential mechanisms that can explain these findings, as well as on the ecological and conservation implications of these.

In general, fGCM levels were more strongly related to proximal than to distal predictors, reflecting that changes in habitat characteristics do not act directly on wildlife’s physiology, but rather through their effects on their behavior. The positive association of travel and the negative associations of rest and fruit consumption with fGCM levels support that physical effort [24,26,66], and reductions in the consumption of high-energy food items (e.g., fruit) [24–27] result in higher levels of fGCMs. These results are well supported given the high-energy demands of spider monkeys associated to their large body size and their high dependency on fruit [33–35], and reflect the energy-mobilizing role of GCs [14–16]. fGCM levels did not differ significantly among sexual status categories, probably because we could not differentiate between cycling and pregnant females.

Consistent with other studies on *Ateles hybridus* [30] and *Canis lupus* [68], we also found that the occurrence of hunting in the vicinity of the groups is a source of stress for spider monkeys. Due to their size and diurnal habits, large primates are very vulnerable to hunting [69]. In Mexico, and particularly in the states of Campeche and Chiapas (where we conducted this study), spider monkeys are taken to black markets to be sold as pets [70]. Therefore, the observed reaction to hunting could indicate that spider monkeys are being poached in our study area. In agreement with this idea, we have observed spider monkeys confined as pets in several houses in the region, and we have received personal communications from local people that agree that this species is poached in the region for pet trade.

Overall, the observed effects of proximal predictors on stress hormone levels would suggest that spider monkeys in fragmented landscapes have higher stress levels than in continuous forests as the literature indicates that: (i) spider monkeys in forest fragments spend less time resting than in continuous forests (see a review of spider monkey’s activity pattern through their geographic range: [71]); and (ii) fruit availability, and their consumption by primates, tends to be lower in forest fragments than in large forest tracts (e.g., [72,73]).

Verifying that fruit availability is a key habitat component for primate stress hormone levels [28,67] we found that fGCM levels were negatively associated with SBAFS, which highlights the importance of preserving fruit sources for primate conservation [49,73]. However, contrary to what we could expect, in our study region, spider monkeys living in smaller tracts of habitat spent less time traveling (year round study: [42]; F4 and F5: Fig 2A). Moreover, these groups have high SBAFSs within their activity areas, and accordingly, fruit consumption did not differ among the study groups regardless of the size of the forest they inhabited. The lower net energy return associated with higher traveling times but similar consumption of energy-rich fruit presented in spider monkeys living in the landscapes with highest forest cover would explain why contrary to what has been observed in other studies with primates [22,24,29,52], in our study, forest cover was positively associated with fGCM levels.

The reason behind why in our study spider monkeys living in larger tracts of forest spent more time traveling compared to those living in smaller forest fragments requires further study. A possible explanation for this is that these groups have more neighboring groups (MA and F1: Table 2), which might force them to spend more time patrolling their home range. Along the same lines, Rimbach et al. [74] suggest that fGCM levels in spider monkeys (*A*. *hybridus*) may increase as a consequence of competition for fruit monopolization, and this competition could be higher in larger tracts of forest with more neighboring groups. As for the lack of differences in fruit consumption among the study groups, this is probably related to the fact that deforestation in the region is relatively recent (< 40 years ago), the remaining forest cover is relatively high (approximately 40%), and the matrix that surrounds the forest fragments is highly heterogeneous; factors that together can contribute to reduce tree species mortality [75]. This is supported by Hernández-Ruedas et al. [75], who also found that in our study region, small forest fragments still harbor similar values of tree basal area and tree species density than the continuous forest.

### Concluding remarks

Our study highlights the complex relationship between habitat transformation processes and primate stress hormone levels, and the usefulness of assessing proximal and distal predictors of stress for obtaining a comprehensive understanding about the effects of habitat disturbance on animal physiology [20]. Unexpectedly, we found that spider monkeys living in smaller forest tracts had lower levels of fGCMs, which we ultimately attributed to the lack of neighboring groups, and relatively high levels of resources probably associated to the recent and moderate degree of deforestation in the region [75]. However, our data suggests that the proximal driver of the relatively lower stress hormone levels of these groups was a higher net energy return due to reduced activity but similar consumption of energy-rich fruit. Finally, we want to emphasize that these results should not be taken as a sign that spider monkeys in forest fragments are not threatened by extinction, as recent studies in the region demonstrate that the future of biodiversity in this region is uncertain (terrestrial mammals: [76]; primates: [31]; plants: [75]; birds: [12]; dung beetles: [77]; amphibians and reptiles: [78]) there is increasing regional support for the production of oil palm (*Elaeis guineensis*) plantations [79], and forest loss is advancing at alarming rates (2.1% of annual loss; [80]). Thus, further studies are required to monitor the health of the remaining populations (e.g., through fGCMs analyses) to obtain a better understanding about the viability of spider monkeys in this biodiversity hotspot, e.g., by relating fGCMs to birth and death rates, intrinsic growth rate, infant survival or related indices.

## Supporting Information

S1 Table Basal areas (m^2^) of tree species used by spider monkeys (*Ateles geoffroyi*) for fruit consumption from six different sites located in the Lacandona rainforest, Mexico. For each site, basal areas were estimated within twenty 50 x 2-m plots (0.2 ha).
(DOC)

S2 Table Linear mixed models (LMMs) with a ΔAIC < 2 examining the effect of proximal and distal predictors of log-transformed fecal glucocorticoid metabolite levels of six groups of spider monkeys (*Ateles geoffroyi*) inhabiting the Lacandona rainforest, Mexico. Samples (nested within groups) and observation days (nested within sampling rounds) were specified as random factors in all models. Marginal *R*^2^ (M *R*^2^) represents the variance explained by fixed factors, and conditional *R*^2^ (C *R*^2^) represents the variance explained by both fixed and random factors. TT = Time traveling, TR = Time resting, DAD = Direct anthropogenic disturbances, TFF = Time feeding on fruit, TFW = Time Feeding on Wood, TFL = Time feeding on leaves, SBAFS = Sum of the basal areas of fruiting-tree species used by spider monkeys for fruit consumption.
(DOC)
