# Supplementary material for: Proximal and Distal Predictors of the Spider Monkey’s Stress Levels in Fragmented Landscapes
Source: PLoS One. 2016 Feb 22;11(2):e0149671. doi: 10.1371/journal.pone.0149671 (PMC4762682; doi:10.1371/journal.pone.0149671)
Supplement: S1 Table — For each site, basal areas were estimated within twenty 50 x 2-m plots (0.2 ha). (DOC) [file pone.0149671.s001.doc]

**S1 Table**. Basal areas (m^2^) of tree species used by spider monkeys (*Ateles geoffroyi*) for fruit consumption from six different sites located in the Lacandona rainforest, Mexico. For each site, basal areas were estimated within twenty 50 x 2-m plots (0.2 ha).

| Family | Tree species | CF | F1 | F2 | F3 | F4 | F5 | Sum |
| --- | --- | --- | --- | --- | --- | --- | --- | --- |
| Moraceae | *Brosimum alicastrum* | 0.476 | 0.945 | 0.297 | 0.935 | 0.876 | 0.237 | 3.766 |
|  | *Brosimum costaricanum* | 0.014 | 0.016 | 0 | 0 | 0 | 0 | 0.03 |
|  | *Brosimum lactescens* | 0.051 | 0.062 | 0 | 0 | 0 | 0 | 0.113 |
|  | *Castilla elastica* | 0 | 0.149 | 0.494 | 0.241 | 0.109 | 0 | 0.993 |
|  | *Ficus* sp. 1 | 0 | 0 | 0 | 0 | 0.016 | 0 | 0.016 |
|  | *Ficus* sp. 2 | 0 | 0 | 0 | 0 | 0.027 | 0 | 0.027 |
|  | *Ficus cotinifolia* | 0 | 0 | 0 | 0.047 | 0 | 0 | 0.047 |
|  | *Ficus insipida* | 0 | 0 | 0 | 0 | 1.745 | 0 | 1.745 |
|  | *Ficus tecolutensis* | 0.534 | 1.82 | 0 | 0 | 1.328 | 0.029 | 3.711 |
|  | *Maclura tinctoria* | 0 | 0 | 0 | 0 | 0 | 0.139 | 0.139 |
|  | *Pseudolmedia oxyphyllaria* | 0.034 | 0 | 0 | 0 | 0 | 0 | 0.034 |
|  | *Trophis mexicana* | 0 | 0 | 0 | 0 | 0 | 0.589 | 0.589 |
|  | *Trophis racemosa* | 0 | 0 | 0 | 0.063 | 0 | 0.401 | 0.464 |
| Sum |  | 1.109 | 2.992 | 0.791 | 1.286 | 4.101 | 1.395 | 11.674 |
| Fabaceae | *Acacia cornigera* | 0 | 0.015 | 0 | 0.044 | 0 | 0.204 | 0.263 |
|  | *Acacia usumacintensis* | 0 | 0 | 0 | 0 | 0 | 0.166 | 0.166 |
|  | *Cojoba arborea* | 0.996 | 0 | 0.262 | 0 | 0 | 0 | 1.258 |
|  | *Dialium guianense* | 1.194 | 1.743 | 0.126 | 1.265 | 0.088 | 0.514 | 4.93 |
|  | *Inga punctata* | 0.273 | 0 | 0 | 0 | 0 | 0 | 0.273 |
|  | *Inga vera* | 0 | 0 | 0 | 0 | 0.082 | 0.215 | 0.297 |
|  | *Lonchocarpus cruentus* | 0 | 0.013 | 0 | 0 | 0 | 0 | 0.013 |
|  | *Lonchocarpus guatemalensis* | 0 | 0 | 0.014 | 0.278 | 0 | 0.134 | 0.426 |
|  | *Pithecellobium arboreum* | 0 | 0.254 | 0 | 0 | 0.012 | 0 | 0.266 |
|  | *Pterocarpus rohrii* | 0.166 | 0.012 | 0 | 0 | 0 | 0 | 0.178 |
| Sum |  | 2.629 | 2.037 | 0.402 | 1.587 | 0.182 | 1.233 | 8.07 |
| Malvaceae | *Guazuma ulmifolia* | 0 | 0 | 0 | 0 | 0 | 0.142 | 0.142 |
|  | *Quararibea funebris* | 0.464 | 0 | 0.019 | 0 | 0 | 0 | 0.483 |
|  | *Quararibea yunckeri* | 0 | 0 | 0.008 | 0 | 0 | 0 | 0.008 |
|  | *Theobroma cacao* | 0.025 | 0 | 0 | 0 | 0.215 | 0.84 | 1.08 |
| Sum |  | 0.489 | 0 | 0.027 | 0 | 0.215 | 0.982 | 1.713 |
| Anacardiaceae | *Mangifera indica* | 0 | 0 | 0 | 0 | 0 | 0.506 | 0.506 |
|  | *Spondias mombin* | 0 | 0 | 0.271 | 0 | 0 | 0.071 | 0.342 |
|  | *Spondias radlkoferi* | 0.289 | 0.027 | 0.291 | 0.041 | 0.053 | 0.378 | 1.079 |
| Sum |  | 0.289 | 0.027 | 0.562 | 0.041 | 0.053 | 0.955 | 1.927 |
| Sapotaceae | *Pouteria durlandii* | 0.164 | 0.015 | 0 | 0.158 | 0.011 | 0 | 0.348 |
|  | *Pouteria campechiana* | 0 | 0.047 | 0.013 | 0.042 | 0 | 0 | 0.102 |
|  | *Pouteria sapota* | 0 | 0 | 0 | 0 | 0 | 0.619 | 0.619 |
| Sum |  | 0.164 | 0.062 | 0.013 | 0.2 | 0.011 | 0.619 | 1.069 |
| Chrysobalanaceae | *Hirtella americana* | 0 | 0 | 0.018 | 0 | 0 | 0 | 0.018 |
|  | *Licania platypus* | 1.423 | 0.765 | 0 | 0 | 0.779 | 0.545 | 3.512 |
| Sum |  | 1.423 | 0.765 | 0.018 | 0 | 0.779 | 0.545 | 3.53 |
| Arecaceae | *Attalea butyracea* | 0 | 0 | 0.076 | 0.193 | 0.128 | 0.127 | 0.524 |
|  | *Sabal mexicana* | 0 | 0.252 | 0.596 | 0.411 | 0 | 0.051 | 1.31 |
| Sum |  | 0 | 0.252 | 0.672 | 0.604 | 0.128 | 0.178 | 1.834 |
| Meliaceae | *Guarea excelsa* | 0 | 0 | 0 | 0.138 | 0.128 | 0 | 0.266 |
|  | *Guarea glabra* | 0.352 | 0.117 | 0 | 0.214 | 0.094 | 0 | 0.777 |
| Sum |  | 0.352 | 0.117 | 0 | 0.352 | 0.222 | 0 | 1.043 |
| Burseraceae | *Bursera simaruba* | 0.198 | 0 | 0.53 | 0.123 | 0 | 0 | 0.851 |
|  | *Protium copal* | 0.022 | 0.043 | 0 | 0.008 | 0.01 | 0 | 0.083 |
| Sum |  | 0.22 | 0.043 | 0.53 | 0.131 | 0.01 | 0 | 0.934 |
| Rubiaceae | *Faramea occidentalis* | 0.008 | 0.012 | 0.025 | 0 | 0 | 0 | 0.045 |
|  | *Psychotria chiapensis* | 0.011 | 0 | 0 | 0 | 0 | 0 | 0.011 |
| Sum |  | 0.019 | 0.012 | 0.025 | 0 | 0 | 0 | 0.056 |
| Ulmaceae | *Ampelocera hottlei* | 0.684 | 0.028 | 0.451 | 0.118 | 0.011 | 0 | 1.292 |
| Sum |  | 0.684 | 0.028 | 0.451 | 0.118 | 0.011 | 0 | 1.292 |
| Icacinaceae | *Calatola laevigata* | 0 | 0.349 | 0.117 | 0.136 | 0 | 0.274 | 0.876 |
| Sum |  | 0 | 0.349 | 0.117 | 0.136 | 0 | 0.274 | 0.876 |
| Lauraceae | *Nectandra ambigens* | 0 | 0.202 | 0.095 | 0.019 | 0.076 | 0 | 0.392 |
| Sum |  | 0 | 0.202 | 0.095 | 0.019 | 0.076 | 0 | 0.392 |
| Clusiaceae | *Garcinia intermedia* | 0 | 0.169 | 0 | 0.188 | 0.01 | 0 | 0.367 |
| Sum |  | 0 | 0.169 | 0 | 0.188 | 0.01 | 0 | 0.367 |
| Boraginaceae | *Cordia bicolor* | 0 | 0.008 | 0.149 | 0 | 0 | 0.092 | 0.249 |
| Sum |  | 0 | 0.008 | 0.149 | 0 | 0 | 0.092 | 0.249 |
| Magnoliaceae | *Talauma mexicana* | 0.236 | 0 | 0 | 0 | 0 | 0 | 0.236 |
| Sum |  | 0.236 | 0 | 0 | 0 | 0 | 0 | 0.236 |
| Polygonaceae | *Coccoloba barbadensis* | 0 | 0 | 0.228 | 0 | 0 | 0 | 0.228 |
| Sum |  | 0 | 0 | 0.228 | 0 | 0 | 0 | 0.228 |
| Combretaceae | *Terminalia amazonia* | 0 | 0 | 0.059 | 0 | 0 | 0 | 0.059 |
| Sum |  | 0 | 0 | 0.059 | 0 | 0 | 0 | 0.059 |
| Annonaceae | *Cymbopetalum mayanum* | 0.018 | 0 | 0.012 | 0 | 0 | 0 | 0.03 |
| Sum |  | 0.018 | 0 | 0.012 | 0 | 0 | 0 | 0.03 |
| Sapindaceae | *Cupania glabra* | 0 | 0 | 0 | 0 | 0.018 | 0 | 0.018 |
| Sum |  | 0 | 0 | 0 | 0 | 0.018 | 0 | 0.018 |
| Urticaceae | *Cecropia obtusifolia* | 0 | 0 | 0 | 0 | 0.01 | 0 | 0.01 |
| Sum |  | 0 | 0 | 0 | 0 | 0.01 | 0 | 0.01 |
| Euphorbiaceae | *Sapium nitidum* | 0 | 0 | 0 | 0 | 0.001 | 0 | 0.001 |
| Sum |  | 0 | 0 | 0 | 0 | 0.001 | 0 | 0.001 |
| Total sum |  | 7.632 | 7.063 | 4.151 | 4.662 | 5.827 | 6.273 | 35.608 |
