# Supplementary material for: Proximal and Distal Predictors of the Spider Monkey’s Stress Levels in Fragmented Landscapes
Source: PLoS One. 2016 Feb 22;11(2):e0149671. doi: 10.1371/journal.pone.0149671 (PMC4762682; doi:10.1371/journal.pone.0149671)
Supplement: S2 Table — Samples (nested within groups) and observation days (nested within sampling rounds) were specified as random factors in all models. Marginal R2 (M R2) represents the variance explained by fixed factors, and conditional R2 (C R2) represents the variance explained by both fixed and random factors. TT = Time traveling, TR = Time resting, DAD = Direct anthropogenic disturbances, TFF = Time feeding on fruit, TFW = Time Feeding on Wood, TFL = Time feeding on leaves, SBAFS = Sum of the basal areas of fruiting-tree species used by spider monkeys for fruit consumption. (DOC) [file pone.0149671.s002.doc]

**S2 Table**. Linear mixed models (LMMs) with a ΔAIC < 2 examining the effect of proximal and distal predictors of log-transformed fecal glucocorticoid metabolite levels of six groups of spider monkeys (*Ateles geoffroyi*) inhabiting the Lacandona rainforest, Mexico.

| Model | AIC | ∆AIC | M *R*^2^ | C *R*^2^ |
| --- | --- | --- | --- | --- |
| Proximal predictors |  |  |  |  |
| TT+TR+DAD+TFF+TFW | 139.82 | 0 | 0.321 | 0.404 |
| TT+RT+DAD | 140.23 | 0.41 | 0.299 | 0.397 |
| TT+TR+DAD+TFF+TFW+TFL | 140.63 | 0.81 | 0.325 | 0.409 |
| TT+TR+DAD+TFL | 140.88 | 1.06 | 0.308 | 0.403 |
| Distal predictors |  |  |  |  |
| Forest cover+SBAFS | 165.7 | 0 | 0.113 | 0.381 |

Samples (nested within groups) and observation days (nested within sampling rounds) were specified as random factors in all models. Marginal *R*^2^ (M *R*^2^) represents the variance explained by fixed factors, and conditional *R*^2^ (C *R*^2^) represents the variance explained by both fixed and random factors. TT = Time traveling, TR = Time resting, DAD = Direct anthropogenic disturbances, TFF = Time feeding on fruit, TFW = Time feeding on wood, TFL = Time feeding on leaves, SBAFS = Sum of the basal areas of fruiting-tree species used by the spider monkey for fruit consumption.
